# Spiritualism and Allied Causes and Conditions of Nervous Derangement

**Published:** 1877-04

**Authors:** 


*Spiritualism and Allied Causes and Conditions of Nervous Derangement.* By WILLIAM A. HAMMOND, M. D., Professor of Diseases of the Mind and Nervous System, in the Medical Department of the University of the City of New York, &c. New York: G. P. Putnam's Sons, 182 Fifth Avenue; 1876; pp. 366. Price, $2 25. Buffalo: Peter Paul & Bro.

This is a bundle of marvelous stories, well told, of events and manifestations supposed, at the time of occurrence, to have been owing to supernatural influences, and between them are sandwiched satisfactory bits of philosophical refutation. They are gathered from a period of time, extending backward from the present, more than two thousand years. Contrasted with jugglers' tricks and professed legerdemain they are shown to be clumsy and inferior. People who were not very credulous would not have been misled by them. Spiritualism, this author regards, as a combination of slight-of-hand deception and nervous disease. Variations of human susceptibility to electricity and magnetism are plainly pointed out; also the power of concentration of attention to produce hypnotism and halluncinations. Certain abnormal mental conditions have long been acknowledged contagious, and, by Dr. Hammond, are said to have been mistakenly named, during some wild religious excitements, and revivals, "An outpouring of the Spirit of God." He calls attention to the lack of substantial proofs of so-called miraculous events, beyond their mere assertion. Stories are quoted from the lives of saints of Romanism and their probable worth is indicated. The different forms of temporary mental aberation are given, with good illustrations; also their self-limitation, power of the will to produce some of them, and the most efficient remedies. He says devils have a decided objection to dwelling in the same body with the bromides.

Hysteria is treated of as an unreasonable, emotional and muscular activity, occurring both with and without suspension of consciousness. The influence of imagination and hope in curing disease or temporarily arresting its ravages is noted. Levitation and incombustibility are the themes of an interesting chapter. That we are surrounded with mysteries which are genuine it is useless to deny; but many things more mysterious are now well understood, and it is almost, if not quite, disreputable to be any longer misled by them. That there still is, in this nineteenth century, need of such an expose, cannot be doubted, but is greatly to be deplored.

